# Mechanical Behavior of Polyethylene Pipes under Strike-Slip Fault Movements

**DOI:** 10.3390/polym14050987

**Published:** 2022-02-28

**Authors:** Lin Li, Liang Qiao, Junming Fan, Yi Zhang

**Affiliations:** 1 School of Petroleum Engineering, China University of Petroleum (East China), Qingdao 266580, China; lilin@upc.edu.cn; 2 Shenzhen Gas Corporation Ltd., Shenzhen 518040, China; lilindaisy@126.com (L.Q.); woshi555666@126.com (J.F.); 3 Shenzhen Engineering Research Center for Gas Distribution and Efficient Utilization, Shenzhen 518000, China; 4 Department of Engineering Mechanics, College of Pipeline and Civil Engineering, China University of Petroleum (East China), Qingdao 266580, China

**Keywords:** PE pipe, strike strip fault, FE simulation

## Abstract

The present paper addresses the mechanical behaviors and failure mechanisms of buried polyethylene (PE) pipes crossing active strike slip tectonic faults based on numerical simulation of the nonlinear response of the soil-pipeline system. The developed finite element (FE) model is first verified through comparing the simulation results with those from large-scale tests and good agreement between simulation and experimental measurements is obtained. The FE model is then applied to investigate the effects of fault crossing angle, pipe and soil properties on the mechanical behavior of PE pipe. The results indicate that the PE pipe crossing negative fault angles is primarily subjected to compression and bending, thus exhibits the phenomenon of buckling. With the increase of crossing angle, there is an increase of the axial strain and the maximum Mises stress in the buckled cross section, and a decrease of the distance between the buckling position and the fault plane. While for positive crossing angles, the PE pipe is mainly subjected to tension and relatively small bending. Increasing the crossing angle causes an increase in bending strain and a decrease in the axial strain. In addition, when the fault moving speed is slower, the axial strain and bending strain are larger, whereas the maximum Mises stress in the buckled cross section and the distance between the buckled position and the fault plane are reduced. Furthermore, the most severe deformation of the pipe is observed when it is buried in the sandy soil, followed by cohesive soil and loess soil.

## 1. Introduction

Use of polyethylene (PE) for natural gas transportation has increased rapidly due to its good physical and mechanical properties and outstanding corrosion resistance. Statistics from Pipeline and Hazardous Materials Safety Administration (PHMSA) shows that over 90% of the newly installed gas pipeline systems are now made of PE. Worldwide, concerns about pipeline safety have grown, amid a boom in natural gas development and in the wake of a string of serious accidents. Factors that can cause failures of PE pipe include excavation damage, natural force damage such as earth movement, landslide and subsidence, material/weld failure, incorrect operation, etc. According to Plastic Pipe Database of American Gas Association, excessive external earth loading is one of the main causes, accounting for 8.4% of PE pipe failures in 2019. Therefore, it is necessary to investigate the mechanical behavior and damage mechanisms of buried PE pipe subjected to earth movement, e.g., earthquake-induced fault movement.

A number of research work have been carried out to examine the influence of permanent ground deformation (PGD) caused by geological disaster on mechanical properties of buried pipelines. Attempts have been made to predict the stress and strain distribution of buried steel pipeline crossing active strike-slip faults using analytical methods since the pioneering work conducted by [1,2] in 1970s. Recently, a series of finite element (FE) models have been developed to evaluate the mechanical response of buried pipeline subjected to PGD. Vazouras et al. investigated the mechanical behavior of buried steel pipelines crossing strike-slip faults using FE simulation, with focus on the effects of soil property, diameter-to-thickness ratio D/t, crossing angle, bend angle and elbow distance [3,4,5,6]. In their work, pipe and soil were modelled using shell element with elastic-plastic model and solid element with the Mohr–Coulomb (M-C) model, respectively. The simulation results suggest that local buckling is the dominant failure mode for buried steel pipe crossing active strike-slip fault with negative crossing angles. In addition, it was concluded that cohesive soils, softer ground conditions, smaller diameter to thickness ratio, smaller pipe internal pressure and X80 steel material (compared with X65) result in a better deformation capacity of the buried pipeline. Similar FE models have been proposed and employed to describe mechanical behavior of steel pipelines crossing strike-slip faults taking boundary conditions into consideration [7], and mechanical behavior of steel pipelines crossing reverse faults [8,9,10] and oblique reverse faults [11]. In addition to the M-C model, the Drucker–Prager (D-P) model was, also, used to analyze mechanical response of steel pipe subjected to strike-slip fault movement with emphasis on the effects of fault modelling [12] and mechanical behavior of steel pipe under reverse fault displacement [13]. In the above FE simulation work, the interaction behavior between pipe and surrounding soil was described using interaction models embedded in the FE software. The other widely used approach is to model pipe–soil interaction by spring element [14,15,16,17,18,19,20].

On the other hand, only a small number of research work have been conducted for the investigation of PE pipe under fault displacement [21,22,23,24,25,26,27]. Xie et al. performed a systematic study on the response of buried PE pipe to strike-slip faulting using shell elements and spring elements to model the behavior of pipe and soil. The results of axial and bending strain distribution from FE simulation were compared with centrifuge tests and effects of fault angle, soil conditions, ratio of buried depth to pipe diameter and pipe diameter on mechanical behavior of buried pipe were investigated. Their results concluded that strike-slip faulting generates larger axial and bending strains with smaller absolute value of negative fault angle, dried sand, larger buried depth to pipe diameter ratio and faster fault movement. Later, the mechanical response of buried high density polyethylene (HDPE) pipe to strike-slip fault displacement was investigated by Robert et al. through using solid elements with M-C model and shell element to describe deformation behavior of soil and pipe, respectively. The numerical results of axial strain were first compared with those from large-scale split box tests and the maximum strain of HDPE pipe was found to deform more substantially when the pipe is buried in courser, wetter and looser soil. It is worth noting that three fault modelling approaches, namely interface, continuous and couple models were proposed and compared for the correct description of discontinuous fault movement. Zhang et al. (2018) analyzed mechanical behavior, i.e., axial stress, axial strain and flattening parameter of PE80 pipe buried in four kinds of soils by modelling soil and pipe with solid elements and shell elements. Their results revealed that loess soil with the weakest cohesion and larger diameter to thickness ratio result in larger strain and flattening parameter of the buried pipe.

Although much effort has been made to elucidate deformation behavior and damage/failure mechanisms of buried pipeline subjected to fault movement, pipe is usually modelled using shell element for computational efficiency. By doing so, the stress and strain distribution along thickness direction cannot be obtained and analyzed, which plays a crucial role during the deformation process of buried pipeline. In addition, mechanical properties of PE pipe are described using a simple hyperbolic constitutive model, which has difficulty in modelling the nonlinear and large deformation behavior of PE materials. Furthermore, few studies compare numerical results with experimental data from large scale tests. As a result, a new FE modelling approach, in which the PE pipe is modelled using a phenomenological constitutive equation and solid elements, is presented in this paper for the investigation of mechanical response of buried PE pipe crossing active strike-slip faults.

## 2. Finite Element Model

**Figure 1:**
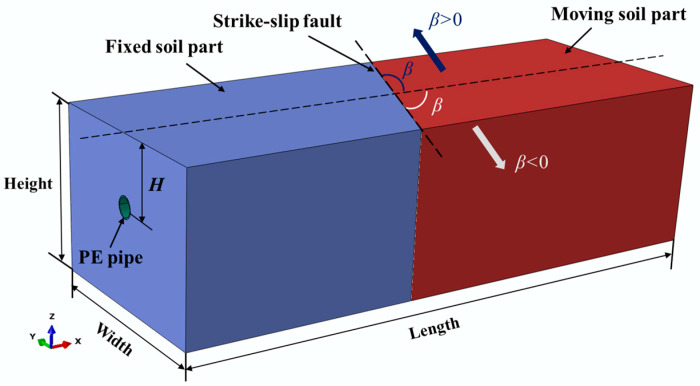
The finite element model of the soil-pipeline system subjected to strike-slip fault movements.

The mechanical response of the PE pipelines subjected to strike-slip fault movements is investigated by the finite element (FE) method using ABAQUS. Figure 1 shows the whole model, including models of the soil and the pipe, where the pipe embedded in the soil along the *x* axis direction. The soil parallelepiped surrounding the pipeline is divided into fixed soil part and moving soil part by the strike-slip fault, oriented at an intersection angle β with respected to the pipe axial axis. For comparison purposes, the dimensions of the proposed FE model are chosen equal to the experimental setup reported in [28]. The PE pipeline, with an external diameter of 400 mm and thickness of 24 mm, is embedded in the soil at a burial depth *H* = 1.22 m, measured from the ground surface to the pipe centerline. The dimensions of the soil parallelepiped are 10.56 m long (the same as the length of the pipeline), 4 m wide and 3 m high.

**Figure 2:**
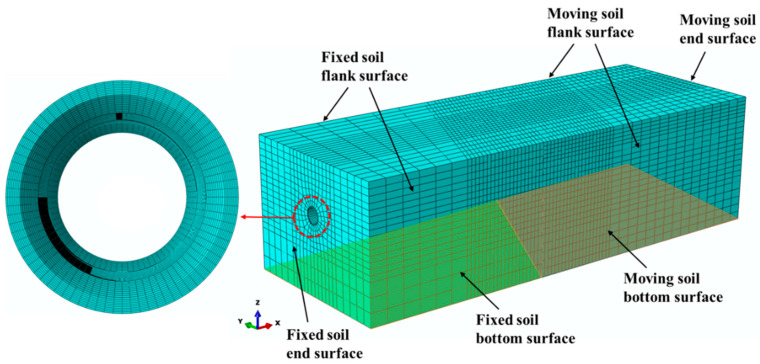
The finite element model of the soil-pipeline system subjected to strike-slip fault movements.

The finite element mesh for the pipeline and soil formation is depicted in Figure 2. For the limit of wall thickness of the shell model, both the pipe and soil are modelled using eight-node reduced-integration solid elements (C3D8R). The mesh for the pipe and soil is finer near the strike-slip fault region where buckling or large deformation is expected to occur. The soil-pipeline interface and the fixed soil and moving soil interface are modeled by the surface–surface contact approach implemented in ABAQUS/Standard, in which Coulomb friction model and separation between two contacting surfaces are available through a penalty function approach. Following suggestions made by [28], the friction coefficient between the soil and PE pipe, between the fixed soil and the moving soil are assigned a value of 0.5, respectively.

The fixed soil part is anchored, which represents the fixed earth ground, and the other one shows the moving ground. The analysis is conducted in two steps: the gravity acceleration is applied first in the Z axis and, subsequently, fault movement is imposed on the moving soil part. The fixed soil bottom and flank surfaces are fixed in X, Y and Z direction, and fixed in Y direction, respectively. The movement of X direction on the end surfaces of fixed soil and pipeline is restricted. The moving soil bottom surface is constrained in the Y and Z direction by setting zero displacements. Moreover, the movement of Y direction on the moving soil flank surface and the movement of X direction of the moving soil and pipe end surfaces are fixed in the first gravity loading step, while are set free in the second loading step. The bottom, flank and end surfaces of moving soil and the end surface of the pipe are displaced forward in a horizontal plane up to the maximum fault offset. The fault crossing angle β is negative when the displacement is applied in negative X and negative Y directions, and positive when the displacement is applied in positive X and positive Y directions.

The constitutive equation proposed by Kwon and Jar [29] and Muhammad and Jar [30] is adopted to describe the large deformation behavior of PE pipe under the strike-slip fault movements. As shown in Equation (1), the constitutive equation is composed of four stress–strain relationships for different strain ranges of elastic-plastic deformation.

(1) $$\sigma (\varepsilon )=\{\begin{array}{ccc} \frac{3}{2(1+\upsilon )}E\varepsilon & \varepsilon \leq \varepsilon_{y} & \left(a\right)\\ d\left\{{[a\left(\varepsilon +b\right)]}^{\left(c-1\right)}-{[a\left(\varepsilon +b\right)]}^{\left(-c\right)}\right\}+e & \varepsilon_{y}\leq \varepsilon \leq \varepsilon_{n} & \left(b\right)\\ \alpha k\varepsilon^{N} & \varepsilon_{n}\leq \varepsilon \leq \varepsilon_{t} & \left(c\right)\\ k\exp\left(M\varepsilon^{\beta }\right) & \varepsilon \geq \varepsilon_{t} & \left(d\right) \end{array}$$

where σ and ε are equivalent stress and equivalent strain, respectively, $\varepsilon_{y}$ the transitional strain from linear to nonlinear deformation, $\varepsilon_{n}$ the critical strain for the on-set of necking and $\varepsilon_{t}$ the strain at the beginning of the exponential hardening. The other parameters (*a*, *b*, *c*, *d*, *e*, *α*, *k*, *N*, *M*, *β*, *A*, *n* and *m*) are user-defined variables for which the values are determined from an iterative process until the results from FE simulation match that from the experimental testing.

The Mohr–Coulomb (M-C) model is used in this paper to quantify the deformation behavior of soil, which is expressed as

(2) $$\tau_{n}=f\left(c,\;\varphi ,\;\sigma_{n}\right)$$

where c and φ are cohesive force and internal friction angle of the soil, respectively. $\sigma_{n}$ is the positive stress applied on the yield surface. The parameters in Equation (2) are the same as those used in [28].

## 3. Results and Discussion

### 3.1. Comparison with Experimental Results

**Figure 3:**
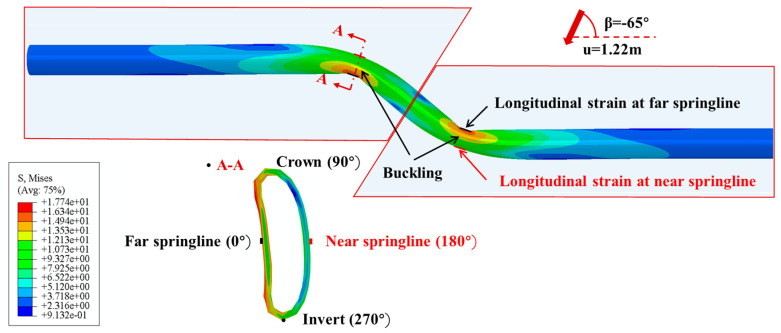
The illustration of the longitudinal strains at the pipe springlines.

The rationality of the FE model is verified through comparing the simulation results with the large-scale test with a fault crossing angle β = −65° reported in [28]. Note that the axial strain is defined as the average of the longitudinal strains at the pipe springlines and the bending strain is obtained as one half the difference between the longitudinal strains at the pipe springlines, as shown in Figure 3.

**Figure 4:**
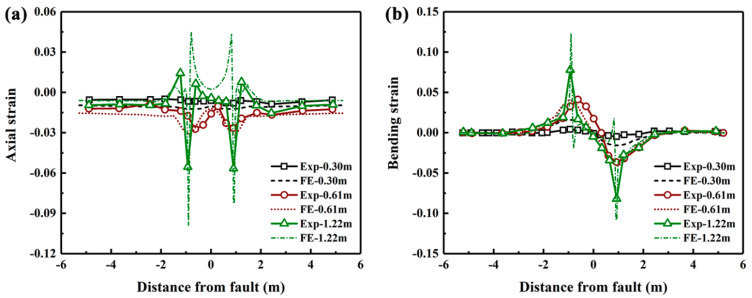
Comparison of the axial and bending strain distributions from the FE simulation with those from the large-scale tests.

**Figure 5:**
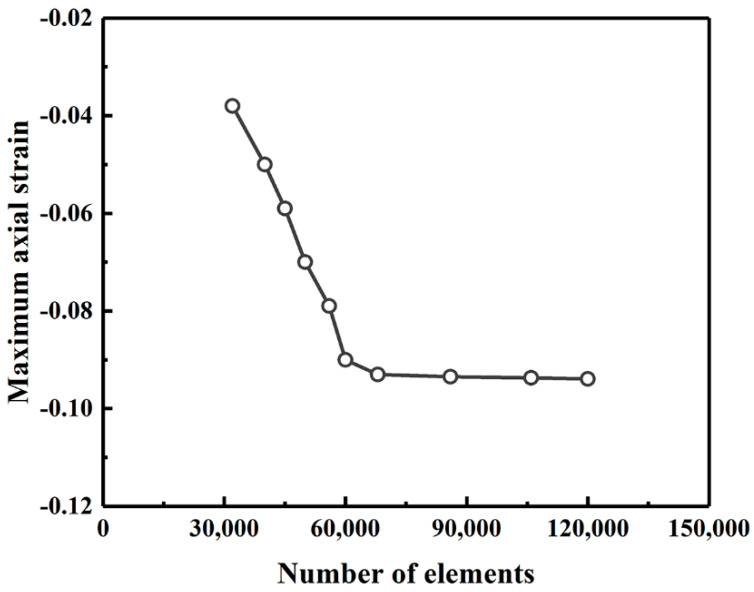
The variation of the maximum axial strain with the number of elements.

Figure 4 compares the axial and bending strain distributions from the FE model with those from the experimental testing at fault displacements of 0.3, 0.61 and 1.22 m. The results show that the simulation results generally match the experimental data. Although the error percentage for the maximum axial can be as large as 56% for the buckled section, the buckling position can be precisely predicted using the proposed FE model. This difference in numerical predictions may due to the limitations of the soil-pipe interaction used in the model, which is not suitable for capturing the post buckling response of the pipeline. Parameters in Equation (1) that are input in the ABABQUS simulation is presented in Table 1. In addition, both the axial and bending strains increases with the increase of fault displacement, and the maximum strains occur at buckling locations, each being 0.91 m away from the fault. Furthermore, the mesh convergence was analyzed through varying the number of the elements. Ten different meshes and their maximum axial strain plot are presented in Figure 5. It can be seen from Figure 5 that the results are converging even after refining the mesh parameters, but as the mesh is refined the time required for the simulation also increases. Therefore, the mesh elements are kept as 68,000 in the following analysis.

**Figure 6:**
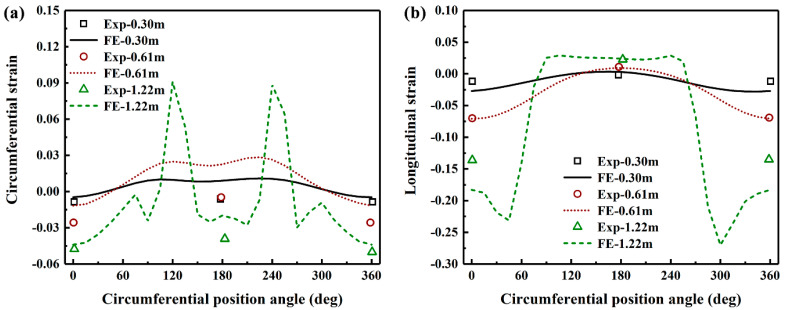
Comparison of the circumferential and longitudinal strain distributions at critical pipe sections where buckling occurs from the FE simulation with those from the large-scale tests.

The strain distribution at critical pipe section where buckling occurs is further investigated using the developed FE model. Figure 6 compares the circumferential and longitudinal strain distributions from the FE simulation with those from the experimental testing at fault displacements of 0.3, 0.61 and 1.22 m. In the large-scale tests, the strain along circumferential and longitudinal direction at far and near springlines (see Figure 3) of buckled cross-section are measured using sensors attached to the pipe. The results show that the predicted results match reasonably well with the experimentally measured data. In addition, both circumferential and longitudinal strains increase with the increase of the fault movement. When the fault offset is 0.3 m, there is little variation of circumferential and longitudinal strains along the circumferential direction, suggesting the pipe deformation is within elastic region. It can be observed from Figure 6b that the maximum longitudinal strain reaches −6.8% (compressive strain) at the circumferential position of 0° (far springline), which suggests that the pipe buckling starts to occur when the fault movement is increased to 0.61 m. With further increase of the fault movement, buckling becomes more severe and the largest compressive longitudinal strain occurs at the circumferential position of 45°.

### 3.2. Effects of Crossing Angle

**Figure 7:**
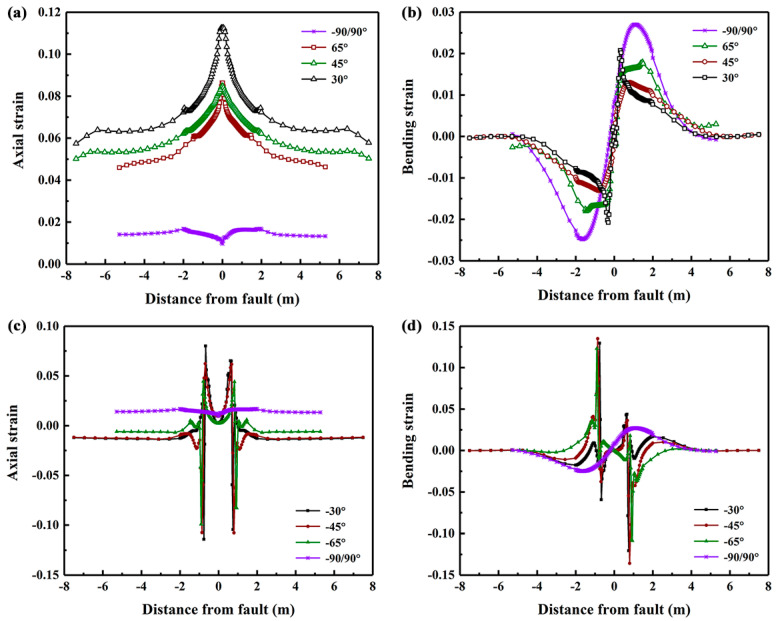
The axial and bending strain distributions in the PE pipeline with various fault crossing angles subjected to fault movement of 1.22 m.

Seven fault crossing angles *β*, i.e., −35°, −45°, −65°, −90°/90°, 65°, 45° and 30° are considered to investigate their influence on the mechanical behavior of buried PE pipelines, as shown in Figure 7. The results show that for positive values of *β*, the axial strain is positive and is much larger than the absolute value of bending strain, indicating that the pipe is primarily subjected to tension and relatively small bending. The axial strain increases with the decrease of crossing angle and the maximum value occurs at the position of fault plane. While the bending strain for cross angle of 90° is the largest and the maximum value occurs at the position that is 1.86 m away from the fault. On the other hand, when the crossing angle is negative, the axial strain at critical positions becomes negative and the absolute values of bending strain are much larger than those in PE pipeline with positive crossing angles, indicating that the pipelines are subjected to bending and compression simultaneously. The peak axial strain increases with the decrease of crossing angle whereas the effect of crossing angle on the bending strain is minor and the maximum bending strain occurs when the crossing angle is −45°.

**Figure 8:**
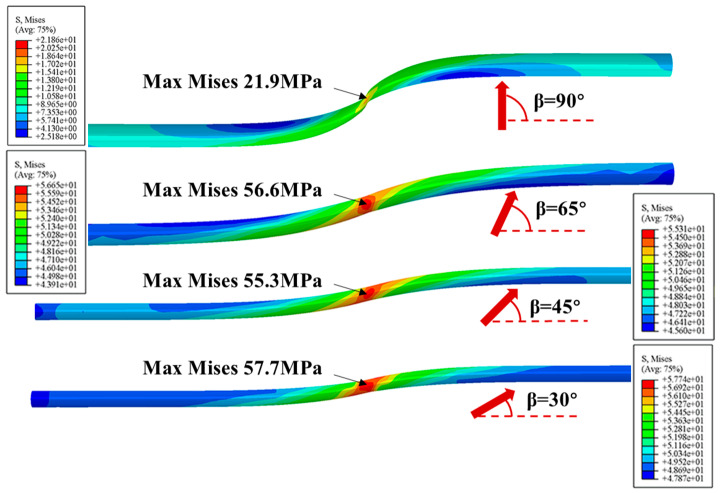
The Mises stress distributions in the PE pipeline with various positive fault crossing angles subjected to fault movement of 1.22 m.

**Figure 9:**
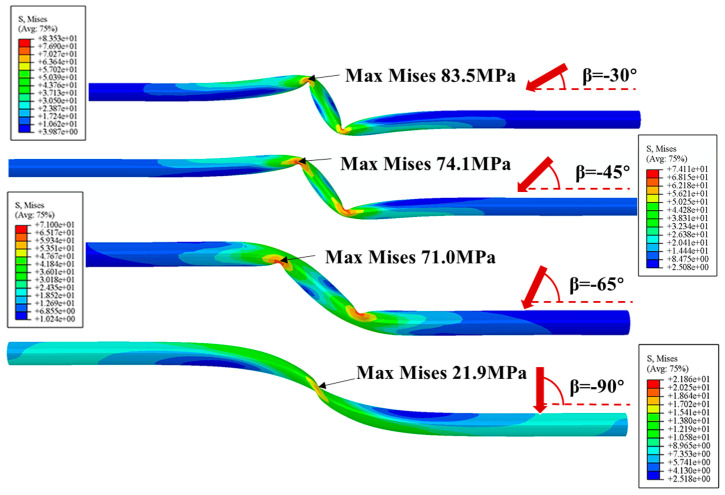
The Mises stress distributions in the PE pipeline with various negative fault crossing angles subjected to fault movement of 1.22 m.

The Mises stress distributions of PE pipeline with positive and negative fault crossing angles subjected to fault movement of 1.22 m are shown in Figure 8 and Figure 9, respectively. The results indicate that the maximum Mises stress occurs at the position of fault plane, and increases with the decrease of crossing angle. The shapes of the deformed pipeline in Figure 8 show that the pipe is primarily subjected to tension and relatively small bending when the crossing angle is smaller than 90°. This phenomenon is consistent with the results presented in Figure 7 that the axial strain for positive crossing angle is positive. On the other hand, when the crossing angle is negative, severe local buckling of pipeline occurs. The results in Figure 9 suggest that the buckling is more severe and the Mises stress is larger when the absolute value of crossing angle is smaller. The maximum Mises stress occurs at the position where the pipe is squeezed by the surrounding soil. In addition, there is one buckling position for crossing angle of −90° while two buckling positions for crossing angles of −30°, −45° and −65°.

**Figure 10:**
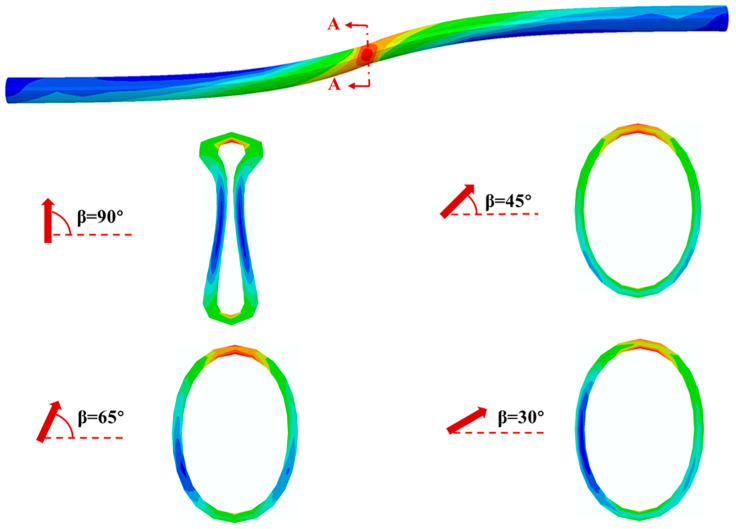
The stress distribution and deformed shapes of critical cross section in the pipeline with various positive fault crossing angles subjected to fault movement of 1.22 m.

Figure 10 shows the deformed shapes and stress distribution of critical cross section in the pipeline with various positive fault crossing angles subjected to the fault movement of 1.22 m. The results show the ovalization of pipe cross section for the crossing angle of 90° is the most obvious whereas the effect of crossing angle on the ovalization for the other three positive angles is relatively small. Since the pipe is modelled using solid element in this study, the stress distribution along the pipe section can be further investigated. The results show that the Mises stress distribution in the critical cross section is not uniform and the maximum Mises stress occurs in the inner wall of the cross section at the crown position.

**Figure 11:**
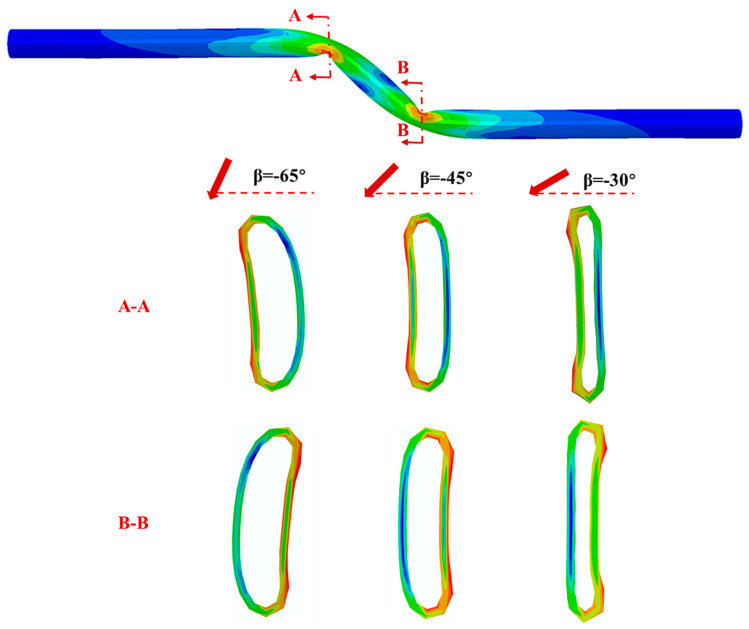
The Mises stress distributions and deformed shapes of critical cross sections in the PE pipeline with various negative fault crossing angles subjected to fault movement of 1.22 m.

Figure 11 presents the deformed shapes and stress distribution of critical cross section in the pipeline with various negative fault crossing angles subjected to the fault movement of 1.22 m. The results show the ovalization of pipe cross section for negative crossing angles is much more severe than positive crossing angles. Since there are two buckling positions in the pipeline, the deformed shapes and stress distribution of cross section A-A (within the fixed soil part) and B-B (within the moving soil part) are analyzed. It can be observed that the pipe cross section for smaller crossing angle is more distorted. The maximum Mises stress occurs at the position close to the crown of the pipe cross section, where the pipe is pushed by the surrounding soil.

### 3.3. Effects of Pipe Property

**Figure 12:**
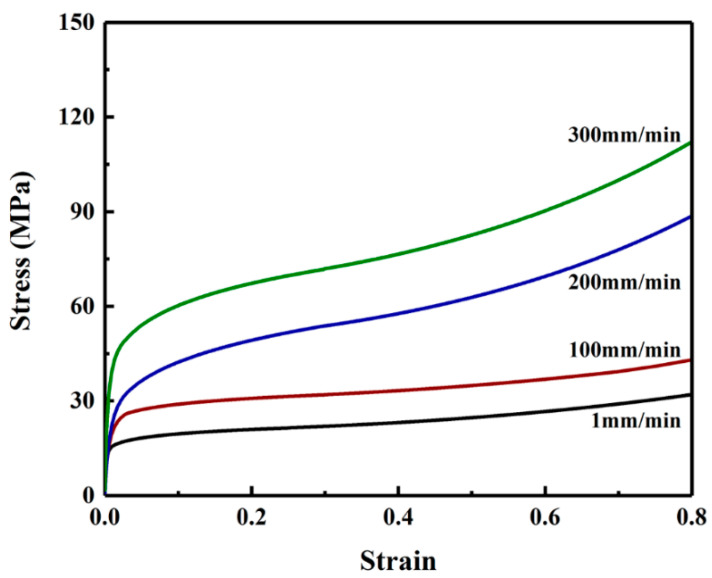
Stress-strain relationships for PE pipe material deformed at speeds of 1, 100, 200 and 300 mm/min.

Due to its viscoelastic and viscoplastic properties, the mechanical behavior of PE material is strongly dependent on the strain rate and temperature. With the increase of strain rate or the decrease of temperature, the PE material will become stronger, but less ductile. The temperature when the fault movement occurs or the speed of the fault movement varies significantly. The influence of pipe property on mechanical response of the buried PE pipe is therefore investigate in this section. The fault is assumed to move at speeds of 1, 100, 200 and 300 mm/min. The stress–strain relationships of PE pipe deformed at these four speeds are described using parameters in Table 1 and depicted in Figure 12.

**Figure 13:**
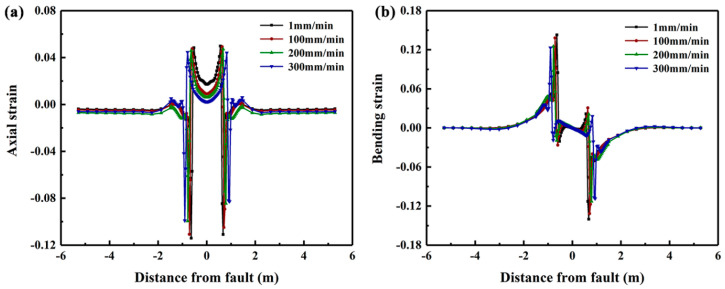
The distribution of axial and bending strains of PE pipeline subjected to fault movement at speeds of 1, 100, 200 and 300 mm/min.

The stress–strain relationships in Figure 12 are inputted in ABAQUS simulation to investigate effects of pipe property on the strain distribution as shown in Figure 13. It should be noted that the crossing angle used in this investigation is −65°. The results show that with the increase of fault moving speed the axial strain and bending strain at buckling position are decreased whereas the effects of fault speed on the positive peak axial strain is relatively small. The axial strain in the pipe region between the two buckling points increases with the decrease of fault moving speed. Furthermore, it can be observed that the distance of the buckling position from the fault plane is increased when the speed is increased.

**Figure 14:**
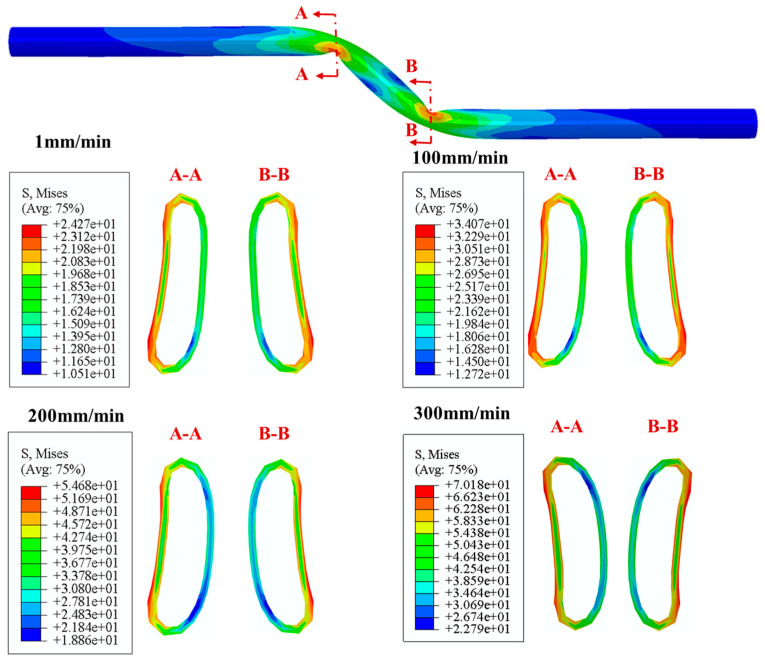
The Mises stress distributions and deformed shapes of critical cross sections in the PE pipeline subjected to strike-slip fault movements with different fault moving speeds.

Figure 14 presents the deformed shapes and stress distribution of critical cross section in the pipeline subjected to the fault movement of 1.22 m at fault moving speeds of 1, 100, 200 and 300 mm/min. The results show that under four fault moving speeds, PE pipelines with crossing angle of −65° exhibits the same failure mode. Additionally, the maximum Mises stress at the buckling cross section increases with the increase of fault moving speed. The influence of fault crossing speed on the deformed shape and ovalization of buckled cross section is relatively small. The maximum Mises in the buckled cross section occurs at the position close to the crown or invert of the contact surface with soil.

### 3.4. Effects of Soil Property

**Figure 15:**
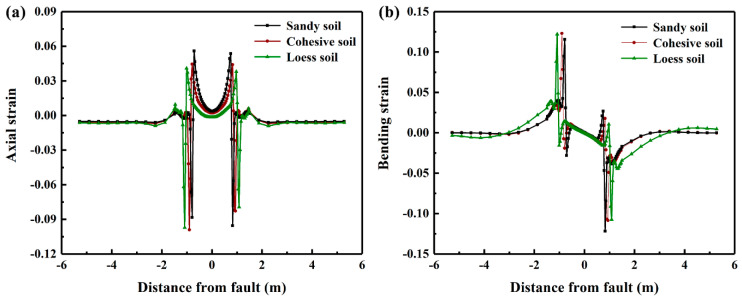
The distribution of axial and bending strains of PE pipeline subjected to fault movement at speeds of 1, 100, 200 and 300 mm/min.

Three kinds of soils, i.e., sandy, cohesive and loess soils are considered to investigate the effects of soil property on the mechanical response of the buried PE pipeline. The soil properties used in this study follow those presented in [24]. The distributions of axial and bending strains of PE pipes buried in various types of soils are summarized in Figure 15. The results indicate that the axial strain at buckling positions of PE pipe buried in sandy soil is the maximum, while the axial strain of PE pipe buried in loess soil is the smallest. On the other hand, the positive bending strain of the PE pipe buried in the fixed loess soil is the largest whereas the negative bending strain of the PE pipe buried in the moving sandy soil in the largest.

**Figure 16:**
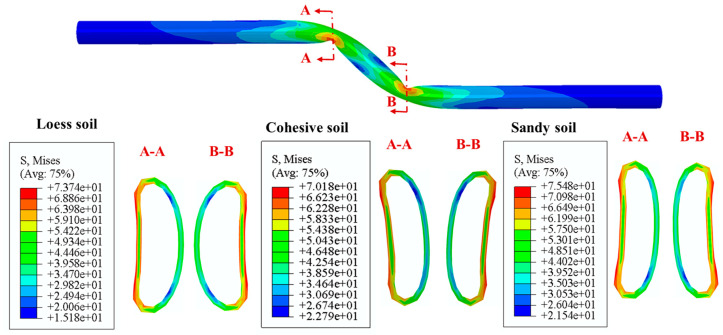
The Mises stress distributions and deformed shapes of critical cross sections in the PE pipeline subjected to strike-slip fault movements with different fault moving speeds.

The deformed shapes and stress distribution of critical cross section in the pipeline buried in various kinds of soils are presented in Figure 16. The results show the ovalization of buckled cross sections is the most severe for the PE pipeline buried in sandy soil, and the distortion of the cross section is the least significant for the pipe buried in loess soil. Furthermore, the maximum Mises stress in the buckled cross sections occurs in the PE pipeline buried in the sandy soil, followed by the loess soil and the cohesive soil.

## 4. Conclusions

The work investigates the mechanical response of PE pipelines subjected to strike slip fault movements. The distribution of axial and bending strains in the pipeline from the proposed FE model are in good agreement with those measured from large scale tests. The FE model is then applied to assess the influence of fault crossing angle, pipe and soil properties on the mechanical behavior of the buried PE pipeline. The results indicate that the deformation and failure modes of the buried PE pipe with positive crossing angles are tension and relatively small bending, while the PE pipe with negative crossing angle is primarily subjected to compression and bending. In addition, it is observed that there are two buckling positions in the PE pipe with negative crossing angles. Furthermore, with an increase of fault moving speed, there is a decreases of axial and bending strains, and an increase of the distance between the buckling position and fault plane. Regarding the effects of soil property, the positive axial strain and negative bending strain are the maximum in PE pipeline buried in sandy soil, followed by cohesive and loess soils.

## Figures and Tables

**Table 1:** Values for parameters and strain range in Equation (1), determined from the FE simulation.

FE Model			PE Pipe under Strike-Slip Fault			
Fault Moving Speed (mm/Min)			1	100	200	300
Equation (1a)		*ε*_y_	0.01	0.005	0.005	0.005
		*E* (MPa)	900	1200	1500	1800
		*ν*	0.4	0.4	0.4	0.4
Equation (1b)		*ε*_n_	0.022	0.02	0.04	0.03
		*a*	9.9	21	69	98
		*b*	0.057	0.025	0.008	0.0039
		*c*	0.05	0.05	0.005	0.001
		*d*	−23.8	−23.8	−23.8	−23.8
		*e*	15.5	15.5	18	33
Equation (1c)		*ε*_t_	0.3	0.3	0.3	0.3
		*αk*	14.52	18.1	80.3	87
		*N*	0.12	0.07	0.26	0.16
Equation (1d)	Section 1	*ε*_t1_	0.6	0.6	0.8	0.8
		*K*_1_	11.7	15.6	53.5	65.6
		*M*_1_	0.68	0.59	0.8	0.8
		*β*_1_	1.8	1.8	1.8	1.8
	Section 2	*ε*_t2_	0.8	0.8	1.0	1.0
		*K*_2_	11.6	14.6	57.5	71
		*M*_2_	0.72	0.71	0.69	0.68
		*β*_2_	1.8	1.8	1.8	1.8
